# Integrated miRNA and mRNA Expression Profiling in Inflamed Colon of Patients with Ulcerative Colitis

**DOI:** 10.1371/journal.pone.0116117

**Published:** 2014-12-29

**Authors:** Jan Van der Goten, Wiebe Vanhove, Katleen Lemaire, Leentje Van Lommel, Kathleen Machiels, Willem-Jan Wollants, Vicky De Preter, Gert De Hertogh, Marc Ferrante, Gert Van Assche, Paul Rutgeerts, Frans Schuit, Séverine Vermeire, Ingrid Arijs

**Affiliations:** 1 Translational Research Center for Gastrointestinal Disorders (TARGID), Department of Clinical and Experimental Medicine, KU Leuven, Leuven, Belgium; 2 Gene Expression Unit, Department of Cellular and Molecular Medicine, KU Leuven, Leuven, Belgium; 3 Translational Cell and Tissue Research, Department of Imaging and Pathology, KU Leuven, Leuven, Belgium; INSERM, France

## Abstract

**Background:**

Ulcerative colitis (UC) is associated with differential colonic expression of genes involved in immune response (e.g. *IL8*) and barrier integrity (e.g. cadherins). MicroRNAs (miRNAs) are regulators of gene expression and are involved in various immune-related diseases. In this study, we investigated (1) if miRNA expression in UC mucosa is altered and (2) if any of these changes correlate with mucosal mRNA expression. Integration of mRNA and miRNA expression profiling may allow the identification of functional links between dysregulated miRNAs and their target mRNA.

**Methodology:**

Colonic mucosal biopsies were obtained from 17 UC (10 active and 7 inactive) patients and 10 normal controls. Total RNA was used to analyze miRNA and mRNA expression via Affymetrix miRNA 2.0 and Affymetrix Human Gene 1.0ST arrays, respectively. Both miRNA and gene expression profiles were integrated by correlation analysis to identify dysregulated miRNAs with their corresponding predicted target mRNA. Microarray data were validated with qRT-PCR. Regulation of *IL8* and *CDH11* expression by hsa-miR-200c-3p was determined by luciferase reporter assays.

**Results:**

When comparing active UC patients *vs.* controls, 51 miRNAs and 1543 gene probe sets gave significantly different signals. In contrast, in inactive UC *vs.* controls, no significant miRNA expression differences were found while 155 gene probe sets had significantly different signals. We then identified potential target genes of the significantly dysregulated miRNAs and genes in active UC *vs.* controls and found a highly significant inverse correlation between hsa-miR-200c-3p and *IL8*, an inflammatory marker, and between hsa-miR-200c-3p and *CDH11*, a gene related to intestinal epithelial barrier function. We could demonstrate that hsa-miR-200c-3p directly regulates *IL8* and *CDH11* expression.

**Conclusion:**

Differential expression of immune- and barrier-related genes in inflamed UC mucosa may be influenced by altered expression of miRNAs. Integrated analysis of miRNA and mRNA expression profiles revealed hsa-miR-200c-3p for use of miRNA mimics as therapeutics.

## Introduction

Ulcerative colitis (UC) is a chronic relapsing inflammatory bowel disease (IBD) and is characterized by a diffuse mucosal inflammation extending proximally from the rectum to a varying degree in the colon [1]. To date, it is widely accepted that IBD is the result of an inadequate and ongoing activation of the mucosal immune system to the luminal microbiota in genetically predisposed subjects. This complex interaction of genetic, immune, and environmental factors causing IBD is reflected in broad gene expression changes which can distinguish IBD from controls. These gene expression changes include genes mainly involved in immune response, cell adhesion, barrier integrity and tissue remodeling [2]–[4].

Gene expression is in part regulated by microRNAs (miRNAs), a group of small (18–25 nucleotides), endogenous, single-stranded non-coding RNA molecules. MiRNAs act as post-transcriptional negative regulators of gene expression by directly binding to the 3′-untranslated regions (UTRs) of specific target mRNAs, which in turn will lead to degradation of the target mRNA or direct repression of the translational process [5].

Aberrant miRNA expression has been linked to carcinogenesis. Evidence suggests that miRNA expression profiles are more discriminating than profiles of protein-coding mRNAs to classify similar human tumor types [6]. Recent studies demonstrated that miRNAs also play an important role in regulating genes involved in immune function [7]. Among these, miR-155-5p and miR-146-5p were first identified as NF-κB regulatory key factors for innate and adaptive immune responses [8], [9]. Since these early reports, it has been demonstrated that a range of miRNAs display changes in their expression levels during inflammation [10]. The evidence that miRNAs are important regulators of innate and adaptive immune responses has encouraged research into the role of miRNAs in immune-related diseases.

In IBD, single nucleotide polymorphisms (SNPs) located at miRNA binding sites affect the expression of target mRNAs involved in disease pathogenesis [11], [12]. Moreover, distinguished miRNA expression profiles have been described in tissues of IBD patients with active and inactive disease [13]–[21]. In this study, we hypothesized that altered expression of miRNAs may be involved in UC pathogenesis and that some specific miRNAs could be potential biomarkers for diagnosis of UC or disease activity. We therefore performed miRNA microarray expression profiling on endoscopy-derived colonic biopsies from patients with active UC, inactive UC and normal control subjects, to investigate the altered miRNA expression in UC colonic mucosa. Gene expression analysis was performed in parallel in a subgroup of UC patients and controls. The results of both miRNA and gene expression profiles were integrated by correlation analysis to identify dysregulated miRNAs with their corresponding predicted target mRNA. This is the first study to correlate whole-genome mRNA and miRNA profiling in UC mucosa. Further studies demonstrated that hsa-miR-200c-3p directly regulates *IL8* and *CDH11* expression. This suggests that miRNAs with altered expression in active UC mucosa regulate the expression of immune- and barrier-related genes.

## Materials and Methods

### Ethics statement

This study was carried out at the University Hospital Gasthuisberg in Leuven. The ethics committee of the University Hospital approved the study (S53684) and all individuals gave written informed consent.

### Patients and biopsy specimens

Colonic mucosal biopsies were obtained during colonoscopy from 17 UC patients and from 10 controls. In UC patients, the biopsies were taken at the most affected sites of the colon. Disease activity was defined using the Mayo endoscopic subscore [22]. In this study, subscore 0 indicated inactive disease, and subscore 2 or 3 as active disease. Ten UC patients had active endoscopic disease and 7 UC patients were in endoscopic remission. Additionally, colonic mucosal biopsies were obtained from 5 active colonic Crohn's disease (CDc) patients and from 5 patients with infectious or ischemic colitis (IC). Similar to UC patients, biopsies were taken at the most inflamed part of the colon but at the edge of the ulcers. Biopsies were immediately placed in RNAlater (Ambion, Austin, TX, USA) and snap-frozen in liquid nitrogen prior to storage at -80°C. Remaining biopsies were used for routine histopathological examination confirming diagnoses of (in)active UC, active CD and ischemic colitis. In (in)active UC patients, histological disease activity was evaluated using the Geboes score [23]. Haematoxylin-eosin stained slides were scored ranging from grade 0 (structural change only) to grade 5 (erosion or ulceration), by a pathologist (GDH) blinded to endoscopic scores. All active UC patients showed a moderate to severe histological score ranging from grade 3.1 to grade 5.4 (median grade 5.2). Six out of 7 UC patients in remission had mild or no structural abnormalities (grade 0.0 or 0.1), and 1 inactive UC patient showed a mild increase of chronic inflammatory infiltrate (grade 1.1). This indicates that the majority of inactive UC patients in this study are in “histologic deep remission”. Infectious colitis (3 cases of *Clostridium difficile* and 1 case of *Entamoeba histolytica*) were confirmed by microbiological analysis of fecal samples. We also studied a control group of 10 individuals with normal colonic mucosa who underwent colonoscopy for colorectal cancer or polyp screening (5 male; median age, 57.7 y; range 51.9–62.6 y). Baseline characteristics of the UC, CDc, IC patients and controls were comparable with regard to sex, age and body mass index (**Table 1**). For continuous variables, two groups were compared according to the Mann-Whitney U-test and using one-way analysis of variance (ANOVA) when more than 2 groups had to be compared. Chi-Square or, when appropriate, Fisher's exact test was used to compare categorical variables. These statistical tests were performed with SPSS software (version 20, Chicago, IL, USA).

**Table 1:** Baseline characteristics.

Baseline characteristic	Active UC (n = 10)	Inactive UC (n = 7)	Active CDc (n = 5)	IC (n = 5)	Controls (n = 10)	p-value
Male/female (%)	6/4 (60.0/40.0)	4/3 (57.1/42.9)	2/3 (40.0/60.0)	1/4 (20.0/80.0)	5/5 (50.0/50.0)	0.64
Median (IQR) age (years)	55.0 (45.3–61.3)	51.1 (36.1–60.4)	45.9 (36.1–60.1)	69.1 (68.2–78.4)	57.7 (51.9–62.6)	0.35
Median (IQR) BMI	27.3 (23.1–29.7)	26.0 (22.7–26.5)	27.1 (20.2–30.4)	21.5 (19.7–22.1)	26.3 (22.4–29.4)	0.46
Median (IQR) duration of disease (years)	7.1 (0.6–20.1)	6.6 (4.0–14.4)	9.8 (7.6–15.0)	NA	NA	0.94
Extent of disease						
left-sided colitis (%)	9 (90.0)	6 (85.7)	NA	NA	NA	1
pancolitis (%)	1 (10.0)	1 (14.3)	NA	NA	NA	1
colitis (%)	NA	NA	4 (80.0)	NA	NA	NA
ileo-colitis (%)	NA	NA	1 (20.0)	NA	NA	NA
Median (IQR) C-reactive protein (mg/dL)	4.1 (2.6–16.2)	1.6 (0.6–2.9)	2.9 (1.5–5.7)	6.1 (5.5–13.2)	NA	0.54
Concomitant medication						
5-Aminosalicylates (%)	9 (90.0)	5 (71.4)	3 (60.0)	NA	NA	0.39
Corticosteroids (%)	4 (40.0)	0 (0.0)	2 (40.0)	NA	NA	0.15
Azathioprine (%)	0 (0.0)	1 (14.3)	0 (0)	NA	NA	0.30
Methotrexate (%)	0 (0.0)	0 (0.0)	1 (20.0)	NA	NA	0.17
Biologics (%)	1 (10.0)	0 (0.0)	0 (0)	NA	NA	0.53
Active smoking (%)	1 (10.0)	2 (42.9)	1 (20.0)	1 (20.0)	NA	0.81
Average (SD) total Mayo score	8 (±1.5)	0 (±0.5)	NA	NA	NA	0.001
Median (IQR) histological Geboes score	5.2 (4.1–5.3)	0.0 (0.0–0.1)	NA	NA	NA	<0.001

IQR: interquartile range; BMI: body mass index; SD: standard deviation.

### miRNA isolation

For miRNA expression analysis, total RNA, including small RNA, was isolated from the biopsies of UC, CDc, IC patients and controls with the mirVana miRNA Isolation kit (Ambion), according to the manufacturer's instructions. The integrity and quantity of total RNA which includes small RNA was assessed with a NanoDrop ND-1000 spectrophotometer (NanoDrop Technologies, Wilmington, DE, USA) and a 2100 Bioanalyzer (Agilent, Waldbronn, Germany) using the Agilent RNA 6000 Nano kit and the Agilent Small RNA kit, respectively. All samples showed good RNA quality (260/280 nm absorbance ratios >1.9 and RNA Integrity Number (RIN)>7).

### miRNA microarray hybridization

To assess miRNA expression in colonic mucosa of active UC, inactive UC patients and controls, total RNA, including a fraction of small RNA, was used and analyzed with Affymetrix GeneChip miRNA 2.0 arrays (Affymetrix, Santa Clara, CA, USA) containing 4560 probe sets for human small RNAs. Out of these probe sets the 1082 probe sets of human mature miRNAs were filtered. These probe sets guarantee 100% coverage of all human mature miRNAs in miRBase v.15 (April, 2010). Probe sets that were deleted in a more recent version of miRBase were excluded for analysis. All steps of the procedure were performed according to the Affymetrix standardized protocol for miRNA 2.0 arrays. Briefly, 250 ng of total RNA was poly-A tailed, followed by ligation of the biotin-labeled molecule, using the FlashTag Biotin HSR RNA Labeling Kit (Genisphere, Hatfield, PA, USA). To verify the labeling efficiency, an ELOSA QC Assay (Genisphere) was run prior to array hybridization, as recommended in the Genisphere protocol. A hybridization cocktail was added to the biotin-labeled RNA sample and heated to 99°C for 5 minutes and then to 45°C for another 5 minutes. This mixture was injected into an Affymetrix miRNA 2.0 array and hybridization was performed under rotation at 48°C for 16 hours. After washing and staining steps using the Affymetrix Fluidics Station, the arrays were scanned on the Affymetrix 3000 GeneScanner. Intensity values for each probe cell (.cel file) were calculated using Affymetrix GeneChip Command Console (AGCC). Quality control of the microarray was performed with the Affymetrix miRNA QC Tool, version 1.1.1.0. The miRNA expression microarray data were deposited according to minimum information about a microarray experiment (MIAME) guidelines to the Gene Expression Omnibus database (series accession number GSE48957).

### mRNA microarray hybridization

For gene expression analysis, total RNA was isolated from adjacent biopsies of a subset of active and inactive UC patients, IC patients and controls using the RNeasy Mini Kit (Qiagen, Valencia, CA, USA), according to the manufacturer's instructions. RNA quality and quantity were assessed with a NanoDrop ND-1000 spectrophotometer (NanoDrop Technologies) and a 2100 Bioanalyzer (Agilent) using the Agilent RNA 6000 Nano kit.

To assess gene expression in colonic mucosa of active UC, inactive UC patients and controls, total RNA (150 ng), without small RNA fraction, was analyzed with Affymetrix GeneChip Human Gene 1.0ST arrays. All steps were performed according to Affymetrix manufacturer's manual 4425209 Rev.B (Affymetrix), and as described earlier by Breynaert *et al.* [24]. Quality assessment and outlier detection was performed before and after normalization using the Bioconductor package arrayQualityMetrics [25]. The mRNA expression microarray data were deposited in MIAME format to the Gene Expression Omnibus database (series accession number GSE48958).

### Microarray expression data Analysis

The microarray data were analyzed as previously described by our group [24], [26], [27]. The Affymetrix raw data (.cel files) were normalized using the robust multichip average (RMA) algorithm [28], obtained from Bioconductor packages in R (version 2.15.0, http://www.r-project.org). GeneChip Human Gene 1.0ST arrays were processed using the R based aroma.affymetrix package (http://www.aroma-project.org/) [29]. The LIMMA package [30] was used for the identification of differentially expressed gene and miRNA probe sets between UC patients and controls. Obtained p-values were adjusted for multiple testing using the false discovery rate (FDR) method of Benjamini-Hochberg (B–H) [31]. Probe sets were considered as biologically significant if showing a >2 fold change (FC) and a FDR<0.05. Probe set annotations of gene probe sets were obtained through the Affymetrix NetAffx website (NetAffx release 33.2, http://www.affymetrix.com/analysis/index.affx) or the NCBI website (http://www.ncbi.nlm.nih.gov). Annotations of miRNA probe sets were derived from the Sanger miRBase database v.20 (June 2013, http://mirbase.org).

Unsupervised hierarchical clustering was applied to the microarray expression profiles, using complete linkage and Euclidian distance as a similarity metric, to visualize similarities among probe sets/samples. Both resulting dendrograms were combined in a two-dimensional heat map with color intensities according to the pattern of gene or miRNA expression. The Bio Functional Analysis tool in the Ingenuity Pathway Analysis program (Ingenuity Systems; http://www.ingenuity.com) was performed to identify the main biological functions associated to the dataset of differentially expressed gene probe sets.

### Integrated analysis of miRNA and mRNA expression

Predicted target mRNAs for the differentially expressed miRNAs were identified using the miRWalk software tool (http://www.ma.uniheidelberg.de/apps/zmf/mirwalk/) which allows simultaneous searches of several prediction programs [32]. Five databases of predicted target mRNAs were selected: miRanda, miRDB, miRWalk, RNA22 and TargetScan. Because no program is consistently superior to all others and in order to reduce the probability of introducing false positives and/or negatives as much as possible, we selected the potential targets that were identified by at least three databases [18], [33]. Predicted targets of some alternative mature miRNAs were only included in miRanda and miRWalk. In these cases, Diana-microT was added as additional database to increase the probability of the target prediction. Only targets predicted by two out of three databases were selected as potential target mRNA of these alternative mature miRNAs.

In a second step we filtered out the mRNAs that were differentially expressed in active UC *vs.* controls in the list of potential targets. Assuming an inverse correlation between miRNA and mRNA expression levels, we selected the dysregulated target mRNAs with an inverse correlation of expression with the respective miRNA [34]. In a last step we specifically focused on groups of genes with a clinical interest in UC pathogenesis and performed the Spearman rank order correlation test to examine correlation relationships between each miRNA and its computationally predicted target mRNA (SPSS, v.20). A significance threshold of 0.05 was assessed to determine the significance of inverse correlation. To correct for multiple testing, the FDR was estimated from the p-values using the method of Benjamini and Hochberg (B–H) [31].

### Quantitative Real-time Reverse-transcription Polymerase Chain Reaction (qRT-PCR) of miRNA

The miRNA microarray expression data was confirmed by performing qRT-PCR for hsa-miR-200c-3p and 10 other differentially expressed miRNAs selected because of their highly significant p-value or fold change (hsa-miR-21-5p, hsa-miR-31-5p, hsa-miR-146a-5p, hsa-miR-155-5p, hsa-miR-196b-5p, hsa-miR-196b-3p, hsa-miR-200b-3p, hsa-miR-375, hsa-miR-422a and hsa-miR-650). Some of these miRNAs have been described earlier in the literature as differentially expressed in UC. Total RNA, including small RNA, from UC, CDc, IC patients and controls, was reversely transcribed into cDNA using the miRCURY LNA Universal RT microRNA PCR System (Exiqon, Vedbaek, Denmark). The reverse transcription was performed using 20 ng of total RNA at 42°C for 60 min followed by heat-inactivation of the reverse transcriptase for 5 min at 95°C. cDNA samples were diluted 1∶80 in nuclease-free water and qRT-PCR was performed in a final reaction volume of 10 µl using SYBR Green master mix and specific LNA miRNA primers (Exiqon), according to the manufacturer's instructions. Samples were run in duplicate on a 7500 Fast Real-Time PCR System (Applied Biosystems, Foster City, CA, USA). The relative amount of each miRNA was calculated as a ratio to the amount of SNORD44, a ubiquitously expressed small nucleolar RNA, using the 2^−ΔΔCT^ method. Results were analyzed using the Mann-Whitney U-test (SPSS, v.20). A p-value of 0.05 was considered significant.

### Quantitative Real-time Reverse-transcription Polymerase Chain Reaction (qRT-PCR) of mRNA

To validate the mRNA microarray data, qRT-PCR was performed for selected differentially expressed mRNAs: target genes cadherin 11 (*CDH11*) and interleukin 8 (*IL8*), 5 differentially expressed mRNAs with a highly significant p-value (*SLC6A14*, *PDZD3*, *AQP8*, *CD55* and *CDH3*) and β-actin, which was used as the endogenous reference gene.

From UC, IC patients and controls, cDNA was synthesized from 500 ng of total RNA using the RevertAid H Minus First Strand cDNA synthesis kit (Fermentas, St. Leon-Rot, Germany). qRT-PCR was performed with the appropriate TaqMan Gene Expression Assay (Applied Biosystems). All assays were run in a final reaction volume of 20 µl using the TaqMan Fast Universal PCR Master Mix (Applied Biosystems) on the Applied Biosystems 7500 Fast Real-Time PCR System. All samples were amplified in duplicate reactions. Relative mRNA expression levels were calculated as 2^−ΔΔCT^ using β-actin as reference mRNA. Results were analyzed using the Mann-Whitney U-test (SPSS, v.20). A p-value of 0.05 was considered significant.

### Cell Culture

Human colonic epithelial HT-29 cells were obtained from ECACC (European Collection of Cell Cultures) and cultured in RPMI-1640 (Lonza, Basel, Switzerland) that was supplemented with 10% fecal bovine serum and 1% penicillin/streptomycin (Invitrogen, Merelbeke, Belgium) at 37°C in a 5% CO_2_ incubator.

### IL8 and CDH11 3′UTR Construct Transfection and Luciferase Reporter Assay

The 3′UTRs of *IL8* (corresponding to 462–1702 nucleotides of RefSeq NM_000584.3) and *CDH11* (corresponding to 2878–3619 nucleotides of RefSeq NM_001797.2) were amplified by PCR from cDNA of HT-29 cells and cloned into the *Xba*I site downstream of the luciferase coding sequence in the pGL3 reporter vector (Promega, Madison, WI, USA). Mutations in the hsa-miR-200c-3p seed regions of the *IL8* and *CDH11* 3′UTRs were generated using the QuikChange II Site-Directed Mutagenesis kit (Agilent), according to the manufacturer's instructions with one modification: LA Taq DNA polymerase (TaKaRa, Kyoto, Japan) was used instead of PfuUltra DNA polymerase. For each miRNA binding site within the wild-type (WT) 3′UTR, 5 nucleotides in the seeding region were substituted. The site-specific mutations were confirmed by DNA sequencing. All used primer sequences are available upon request.

HT-29 cells were plated in 24-well dishes overnight before transfection (1.5×10^5^ cells per well). Cells were transfected with luciferase reporter constructs (500 ng/well) using Lipofectamine 3000 (Invitrogen) according to the manufacturer's protocol. To correct for transfection efficiency, 25 ng of a β-galactosidase expression plasmid (pCMV-β-gal; Agilent) was used as an internal control. Forty-eight hours after transfection, cells were lysed in 75 µl Passive Lysis Buffer (Promega) and luciferase activities of the cell lysates were measured in a Luminoskan Ascent luminometer (Thermo Labsystems, Franklin, MA, USA) using the Promega Luciferase Assay Reagent. β-galactosidase activities of the same samples were measured using the Galacto-Light Chemiluminescent Reporter Gene Assay System (Tropix Inc., Bedford, MA, USA). Each transfection assay was performed in triplicate and data represents three independent experiments. The luciferase value of each sample was normalized to its β-galactosidase activity. Differences in luciferase activity were analyzed by 2-tailed Student's *t* test and a p-value of 0.05 was considered significant (SPSS, v.20).

### Transfection of miRNA Inhibitor

HT-29 cells were plated into 24-well dishes at 1.5×10^5^ cells and incubated overnight. Various concentrations (5, 10 or 20 nM) of hsa-miR-200c-3p LNA Power Inhibitor (anti-miR-200c-3p) or negative control LNA Power Inhibitor (Exiqon) were transfected after 24 hours using Lipofectamine 3000 (Invitrogen) and the transfected cells were incubated for 48 hours. Total RNA, including miRNA, was isolated from the cells (see paragraph ‘miRNA isolation’) and stored at -80°C for qRT-PCR analysis of miR-200 family members hsa-miR-200b-3p and hsa-miR-429.

In a second series of experiments, HT-29 cells were co-transfected with luciferase vectors (original luciferase vector, luciferase vector containing WT 3′UTR, or luciferase vector containing mutant 3′UTR), 10 nM of either anti-miR-200c-3p or negative control, and pCMV-β-gal as internal control, using Lipofectamine 3000 (Invitrogen). After 48 hours, luciferase and β-galactosidase activity were measured as described above. Each transfection assay was performed in triplicate and data represent three independent experiments. Differences between groups were determined using 2-tailed Student's *t* tests (SPSS, v.20). A p-value of 0.05 was considered significant.

## Results

### 1. Differentially expressed miRNAs in UC vs. controls

**Figure 1:**
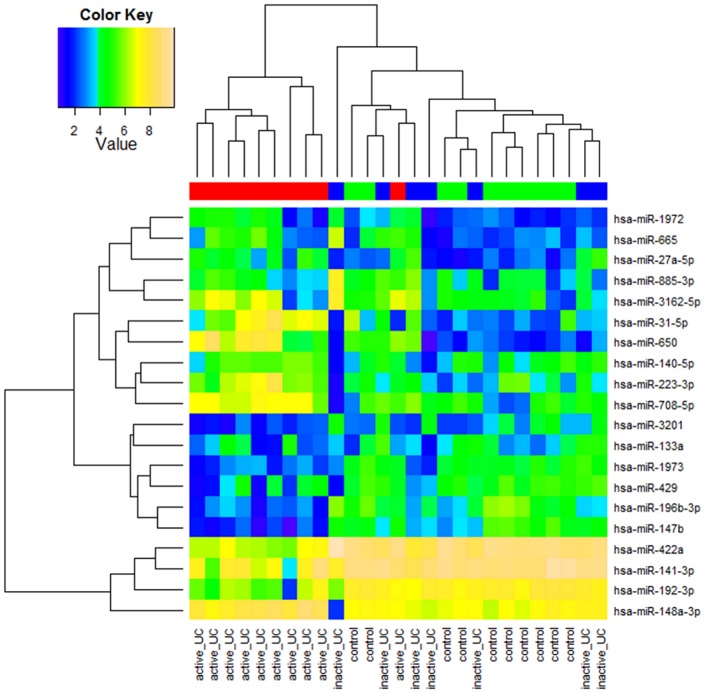
Heatmap of miRNA expression in mucosal colonic biopsies of UC patient and control cohorts. Unsupervised hierarchical clustering of all samples based on the log2 expression values of the top 20 most variable miRNAs. Samples are shown in the columns and miRNAs in the rows. The boxes in color indicate the log2 intensities of the miRNAs, with blue indicating low expression and yellow indicating high expression.

Unsupervised cluster analysis of the top 20 probe sets encoding miRNAs with the highest variation in expression across the 27 samples clearly identified two major clusters (**Fig. 1**). Cluster I included all the controls, all 7 inactive UC patients and only 1/10 active UC patient, and cluster II contained 9/10 active UC patients. These findings indicate distinctive miRNA expression profiles based on colonic inflammatory load.

**Figure 2:**
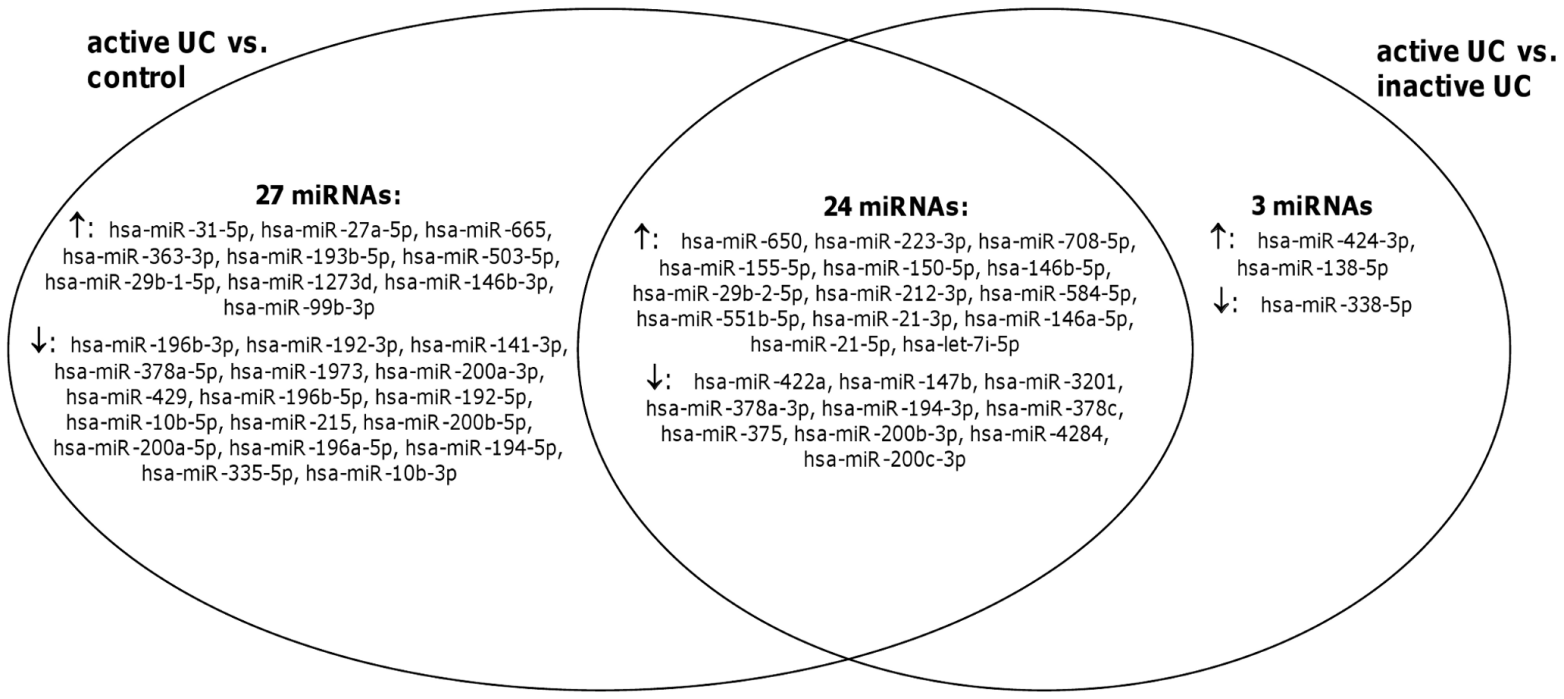
Venn diagram of the overlap of miRNA profiles in comparative analyses between (in)active UC and controls. The differentially expressed miRNAs in the comparative analyses active UC *vs.* controls and active UC *vs.* inactive UC are depicted in two overlapping circles. An overlap of 24 miRNAs (14 up- and 10 downregulated) was observed between both analyses.

MiRNA expression profiles were then compared between the different groups. If we compared the miRNA expression profiles of all UC patients *vs.* normal controls, four mature miRNAs were significantly differentially expressed: hsa-miR-675-5p was significantly upregulated in UC *vs.* controls, while hsa-miR-378a-5p, hsa-miR-196b-5p and hsa-miR-10b-5p were significantly downregulated. We identified 51 (24 up- and 27 downregulated) mature miRNAs that were significantly differentially expressed in active UC *vs.* controls (**S1 Table**), while no significant differences were observed between inactive UC and controls. Comparison of the miRNA expression profiles between active UC and inactive UC identified 27 miRNAs (16 up- and 11 downregulated) that were significantly different, with 24 miRNAs common to the significant miRNAs between active UC and controls (**Fig. 2**). These results substantiate the different clusters observed by the unsupervised clustering analysis, suggesting distinctive miRNA expression profiles based on inflammatory activity.

### 2. Differentially expressed mRNAs in UC vs. controls

**Figure 3:**
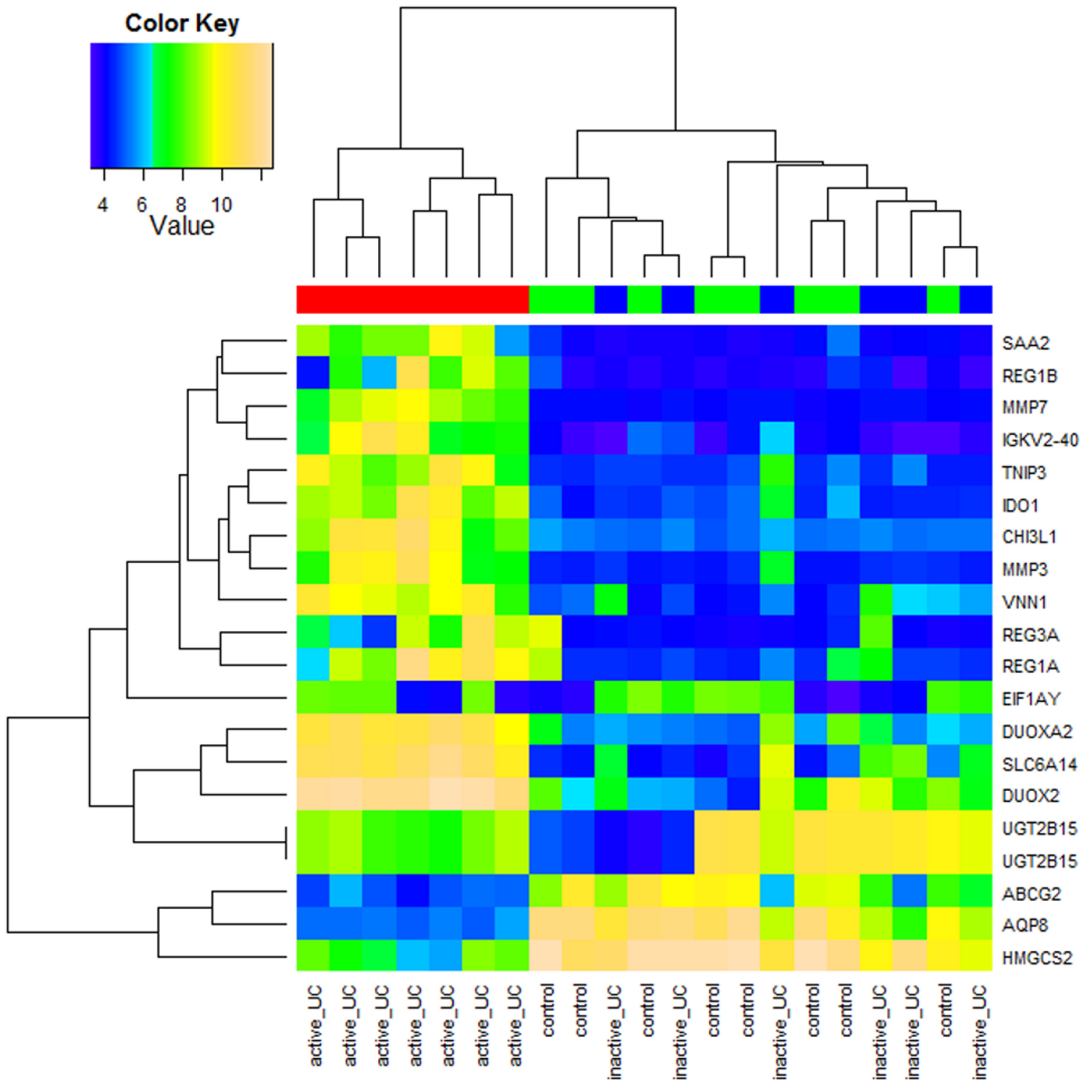
Heatmap of mRNA expression in mucosal colonic biopsies of UC patient and control cohorts. Unsupervised hierarchical clustering of all samples based on the log2 expression values of the top 20 most variable mRNAs. Samples are shown in the columns and mRNAs in the rows. The boxes in color indicate the log2 intensities of the mRNAs, with blue indicating low expression and yellow indicating high expression.

We also investigated the mRNA expression profile of a subset of the same patients for which the miRNA expression profile was examined (7 active UC, 6 inactive UC patients and 8 controls). Unsupervised cluster analysis of the top 20 probe sets encoding mRNAs with the highest variation in expression across the 21 samples showed two major clusters (**Fig. 3**). Cluster I includes all the active UC patients, while cluster II contains all inactive UC patients and all controls. Comparable with the findings of the miRNA expression data, this indicates distinctive mRNA expression profiles based on degree of inflammation.

Second, mRNA expression profiles of active UC, inactive UC patients and controls were compared. In active UC *vs.* controls, we identified 1543 significantly differentially expressed probe sets (976 up- and 567 downregulated) representing 1288 annotated genes (**S2 Table**). After comparison of inactive UC *vs.* controls, 155 significantly differentially expressed gene probe sets (73 up- and 82 downregulated) were observed. Between active UC and inactive UC 991 gene probe sets (796 up- and 195 downregulated) were significantly different (**S1 Fig.**). These results substantiate the different clusters observed by the unsupervised clustering analysis, suggesting distinctive miRNA expression profiles based on inflammatory activity.

A Bio-Functional Analysis tool in the IPA program was performed on the differentially expressed gene probe sets from the comparative analysis between active UC patients and controls to identify which biological functions are associated. The mRNAs encode proteins that were predominantly involved in the following immune-related biological functions: cellular movement, immune cell trafficking, hematological system development and function, cell-to-cell signaling and interaction, and humoral immune response (**S2 Fig.**). In contrast, the differentially expressed gene probe sets from the comparison between inactive UC and controls encode proteins that are associated with cellular metabolism (lipid metabolism, cellular development, connective tissue development and function. **S3 Fig.**).

### 3. Correlation between expression levels of significantly differentially expressed miRNAs and their predicted target mRNAs

In order to identify target mRNAs of one or more of the differentially expressed miRNAs which potentially play a role in UC pathogenesis, we applied two selections of the potential targets. The target mRNAs should be: (1) differentially co-expressed together with the altered miRNAs in the inflamed colonic mucosa of UC patients; and (2) have an inverse correlation of expression with the miRNA by which it is targeted. This resulted in 3328 pairs of significantly altered miRNAs and predicted target mRNAs with an inverse correlation of expression in active UC *vs.* controls (**S3 Table**). More than 60% (806/1288) of the altered annotated genes are potentially regulated by one or more of the 51 altered miRNAs in active UC *vs.* controls. The Bio-Functional Analysis tool in the IPA program was used to assign biological functions to the putative target mRNAs. The mRNAs encode proteins that were predominantly involved in the biological functions: cellular movement, immune cell trafficking, hematological system development and function, tissue morphology and cellular development (**S4 Fig.**). All of these miRNA-regulated biological pathways are related to the pathogenesis of UC.

**Figure 4:**
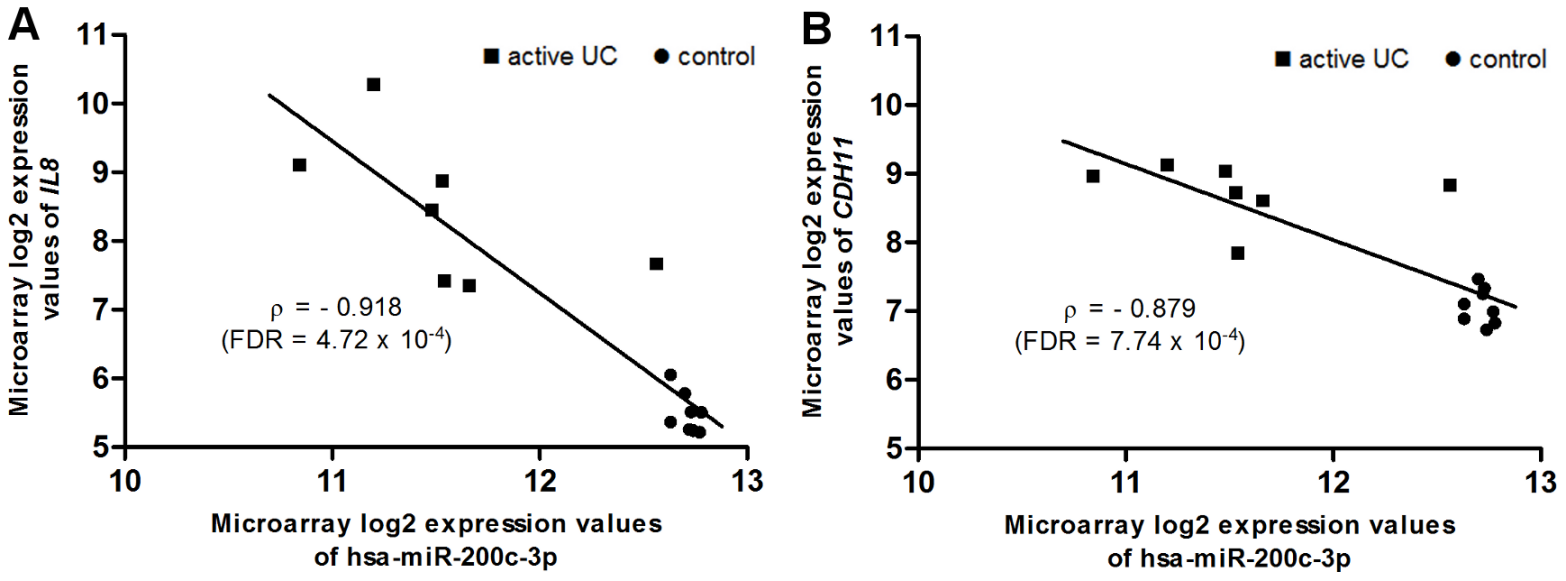
Scatter plot of expression levels of hsa-miR-200c-3p and its predicted target genes. Correlation between hsa-miR-200c-3p expression values and *IL8* (A) and *CDH11* (B) expression values, with their respective Spearman correlation coefficient.

Of all miRNA-mRNA pairs, we selected the genes with an interesting role in UC pathogenesis: UC susceptibility genes [35], anti-microbial peptides [2], cell-adhesion molecules [3] and genes related to the intestinal epithelial barrier function (**S4 Table**). Hence, we ended up with a list of 357 miRNA – target mRNA pairs with clinical interest in UC. We detected a significant correlation in 318 miRNA-mRNA pairs (**S5 Table**). Four of the top 10 most significantly inversely correlated miRNA – target mRNA pairs are related to one specific miRNA, hsa-miR-200c-3p (**Table 2**). This miRNA was chosen to validate using qRT-PCR, together with two target mRNAs of interest: *IL8*, a UC susceptibility gene, and *CDH11*, a gene related to intestinal barrier function (**Fig. 4**).

**Table 2:** Top 10 most significantly correlated miRNA-mRNA pairs.

miRNAs	Predicted target		Spearman correlation coefficient	p-value	FDR
hsa-miR-200c-3p	*IL8*	UC susceptibility gene	−0.918	1.42×10^−6^	4.72×10^−4^
hsa-miR-200c-3p	*LRRK2*	UC susceptibility gene	−0.904	3.88×10^−6^	6.46×10^−4^
hsa-miR-200c-3p	*CALU*	UC susceptibility gene	−0.893	7.49×10^−6^	7.46×10^−4^
hsa-miR-200a-5p	*FADS2*	UC susceptibility gene	−0.889	9.19×10^−6^	7.46×10^−4^
hsa-miR-141-3p	*IKZF1*	UC susceptibility gene	−0.886	1.12×10^−5^	7.46×10^−4^
hsa-miR-200c-3p	*CDH11*	Barrier gene	−0.879	1.63×10^−5^	7.74×10^−4^
hsa-miR-200b-5p	*CXCL9*	Cell adhesion molecule	−0.879	1.63×10^−5^	7.74×10^−4^
hsa-miR-141-3p	*PRDM1*	UC susceptibility gene	−0.875	1.95×10^−5^	7.74×10^−4^
hsa-miR-194-5p	*FADS2*	UC susceptibility gene	−0.871	2.32×10^−5^	7.74×10^−4^
hsa-miR-378a-5p	*FADS2*	UC susceptibility gene	−0.871	2.32×10^−5^	7.74×10^−4^

### 4. Validation of the microarray expression data by qRT-PCR

**Figure 5:**
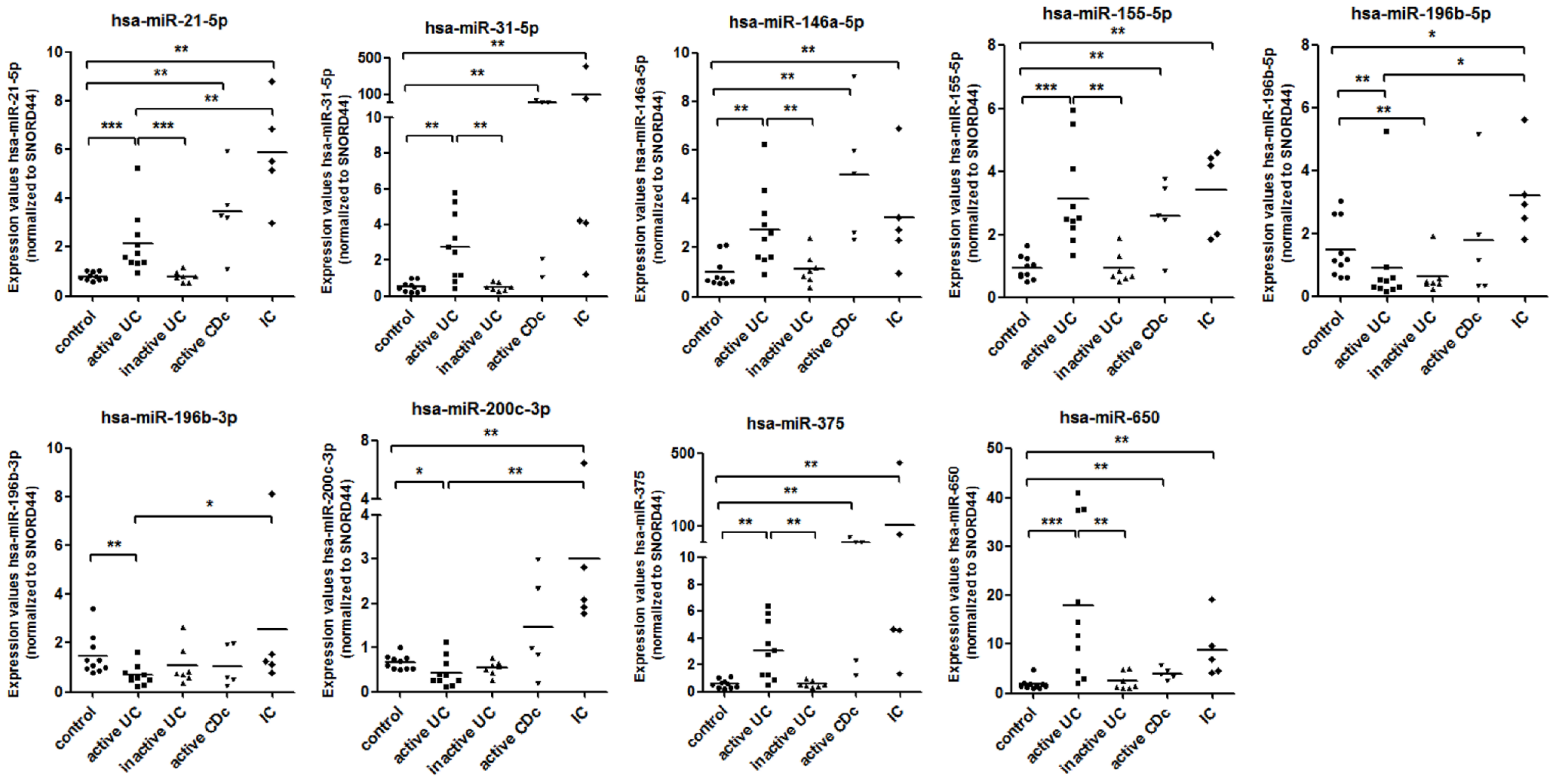
Validation of expression levels of 10 selected miRNAs in human colon. Boxplots of the expression level of hsa-miR-200c-3p and 9 other significantly differentially expressed miRNAs in controls (n = 10), active UC (n = 10), inactive UC (n = 7), active CDc (n = 5) and IC (n = 5) patients, as assessed by qRT-PCR (line, average; *p<0.05; ** p<0.01; *** p<0.001).

Based on the correlation analysis of miRNA and mRNA expression, the significance levels of miRNA and mRNA microarray data and previous literature, 11 miRNAs and 7 mRNAs were chosen to confirm their altered expression in active UC compared to controls by qRT-PCR. Consistent with the microarray data, we were able to confirm that 5 miRNAs (hsa-miR-21-5p, hsa-miR-31-5p, hsa-146a-5p, hsa-miR-155-5p and hsa-miR-650) were significantly increased in active UC mucosa, whereas 3 miRNAs (hsa-miR-196b-5p, hsa-miR-196b-3p and hsa-miR-200c-3p) were significantly decreased, as compared with controls. Remarkably, although initially identified in the microarray analysis as significantly downregulated, we identified a significant upregulation of hsa-miR-375 in active UC patients *vs.* controls. Relative expression levels of these 9 miRNAs are depicted in Fig. 5. qRT-PCR did not confirm the differential expression of hsa-miR-200b-3p in active UC compared to controls. Expression of hsa-miR-422a was undetectable in all samples (data not shown). In inactive UC mucosa, all miRNAs with altered expression in active UC had expression levels comparable to the levels in normal controls, except for 1 miRNA: hsa-miRNA-196b-5p. Similarly to active UC, expression of this miRNA was downregulated in inactive UC compared to controls. To assess the specificity of the miRNA expression alterations in UC tissue, we determined the expression of 9 miRNAs in mucosa of patients with active CDc and IC. All 6 miRNAs (hsa-miR-21-5p, hsa-miR-31-5p, hsa-146a-5p, hsa-miR-155-5p, hsa-miR-375 and hsa-miR-650) with increased expression in active UC demonstrated a similar upregulated expression profile in both active CDc and IC compared to healthy controls. However, 2 miRNAs (hsa-miR-196b-5p and hsa-miR-200c-3p) with decreased expression in active UC were significantly upregulated in IC compared to both active UC and controls. These results indicate that the miRNA expression profile of active UC mucosa is distinct from inactive UC, IC and normal controls. Although, we could not identify significant differences in miRNA expression between active UC and CDc, indicating a similar miRNA expression profile in inflamed colonic mucosa in IBD patients.

**Figure 6:**
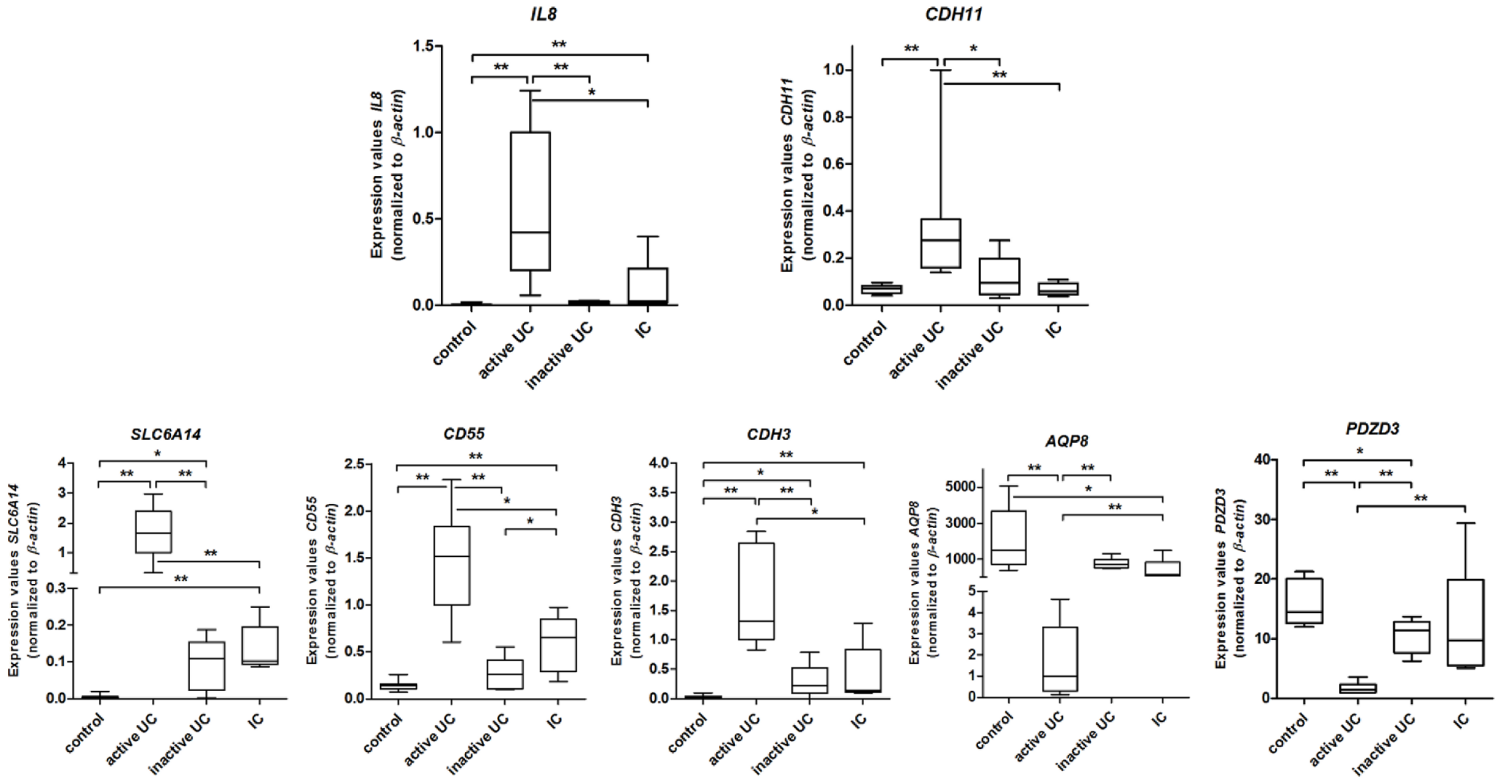
Validation of expression levels of 7 selected mRNAs in human colon. Boxplots of the expression level of *IL8*, *CDH11*, *SLC6A14*, *CD55*, *CDH3*, *AQP8* and *PDZD3* in controls (n = 8), active UC (n = 7), inactive UC (n = 6) and IC (n = 5) patients, as assessed by qRT-PCR (box, 25%–75%; whisker, 5%–95%; *p<0.05; ** p<0.01).

Differential expression of all 7 mRNAs could be confirmed. Expression of *IL8*, *CDH11*, *SLC6A14*, *CD55* and *CDH3* was significantly increased, while *AQP8* and *PDZD3* were significantly decreased in active UC compared to inactive UC and controls. In inactive UC, expression of *SLC6A14*, *CDH3* and *PDZD3* was significantly different from controls. To check if the 7 gene expression alterations are a marker for inflammation or specific for active UC, gene expression was also measured in the biopsies of IC patients. As compared to controls, we identified a significant upregulation of *IL8*, *SLC6A14*, *CD55* and *CDH3* and a significant downregulation of *AQP8* in IC patients. The fold change in expression of these 5 genes is significantly smaller in IC patients than in active UC patients, compared to controls. This suggests that the alteration in expression of these 5 genes probably reflect unspecific inflammation rather than events specific for UC. However, expression of *CDH11* and *PDZD3* is not significantly different between IC patients and controls. This indicates that alterations in expression of *CDH11* and *PDZD3* are part of a specific signature of active UC (**Fig. 6**).

### 5. Regulation of IL8 and CDH11 by hsa-miR-200c-3p in colonic epithelial cells

**Figure 7:**
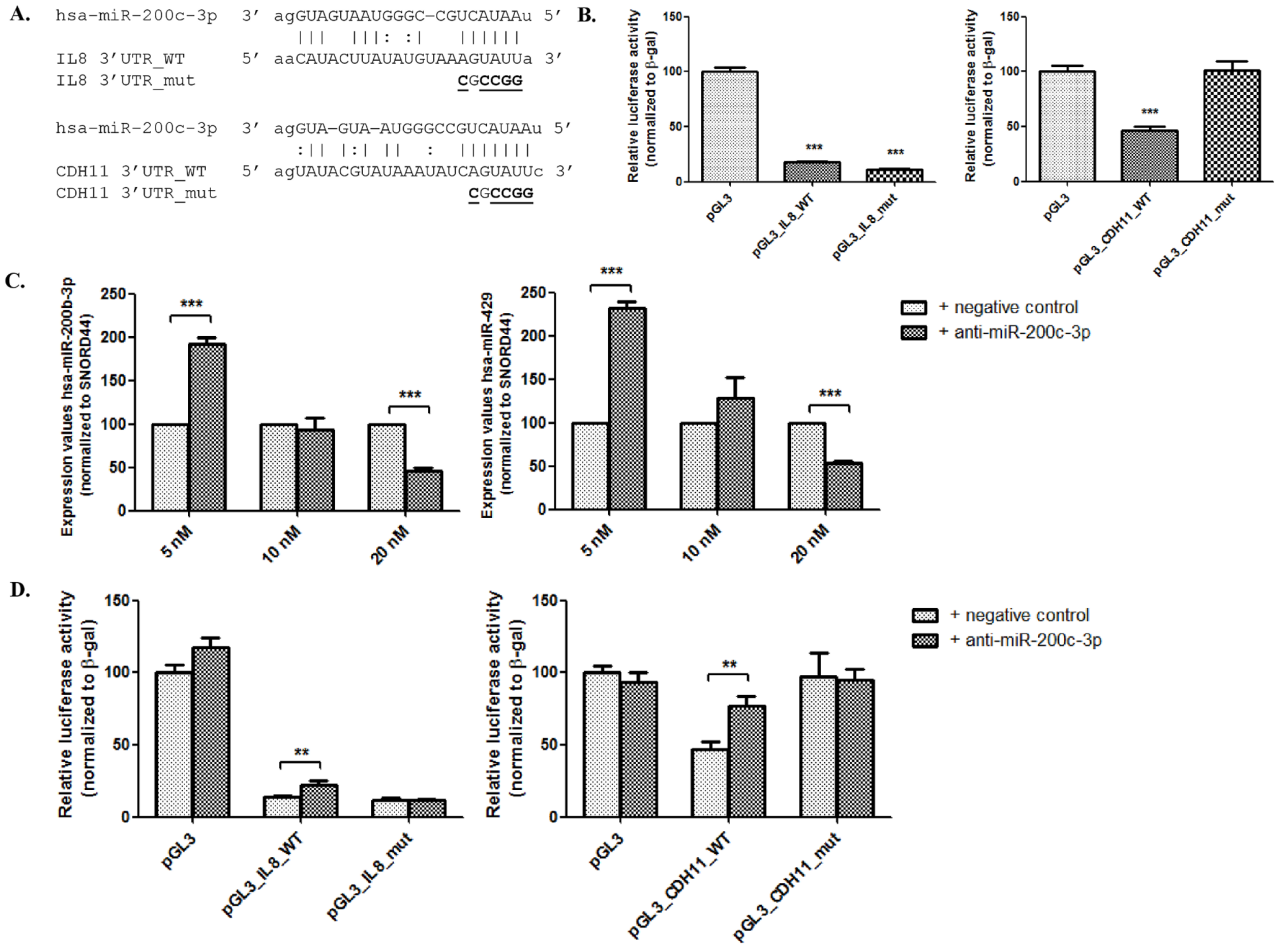
Luciferase reporter assay showing that hsa-miR-200c-3p regulates *IL8* and *CDH11* expression in HT-29 cells. (A.) Sequence alignments of hsa-miR-200c-3p with the wild-type and mutant 3′UTR of target mRNAs *IL8* and *CDH11*, cloned into the pGL3 vector. Mutated nucleotides are underlined (B.) The original pGL3 vector, vectors with wild-type 3′UTRs and vectors with mutant 3′UTRs were transfected into HT-29 cells and luciferase activity (normalized to β-galactosidase activity) was quantified 48 h after transfection. (*** p<0.001, compared to pGL3-vector). (C.) Co-transfection of 10 nM of anti-miR-200c-3p or negative control inhibitor with luciferase reporter vectors in HT-29 cells (** p<0.01, compared to pGL3-vector with negative control).

Correlation analysis suggested a highly significant negative correlation between expression levels of hsa-miR-200c-3p and its potential target mRNAs of interest: *IL8* and *CDH11*. To determine whether these mRNAs are targeted by endogenous miRNAs, we transfected HT-29 cells with luciferase reporter vectors containing the WT 3′UTR (pGL3_IL8_WT and pGL3_CDH11_WT with putative binding site for hsa-miR-200c-3p) or luciferase vector containing mutant 3′UTR (pGL3_IL8_mut and pGL3_CDH11_mut with mutations at the binding site) (**Fig. 7A**). Compared to the original luciferase vector (pGL3), transfecting the wild-type constructs pGL3_IL8_WT and pGL3_CDH11_WT resulted in >80% and >50% reduction in relative luciferase activity, respectively (p<0.001) (**Fig. 7B**). Mutating the binding site in the 3′UTR of *CDH11* resulted in a restitution of the luciferase activity to the level of the original vector. This indicates that endogenous hsa-miR-200c-3p can target the 3′UTR of *CDH11* and hereby regulating its expression. However, mutations in the binding site of 3′ UTR of *IL8* did not restore the luciferase activity (**Fig. 7B**). Endogenous miRNAs, excluding hsa-miR-200c-3p, can suppress the luciferase activity by targeting the 3′UTR of *IL8* at different binding sites.

Hsa-miR-200c-3p is part of the miR-200 family, where it shares the same seed sequence with hsa-miR-200b-3p and hsa-miR-429. If a single miRNA is inhibited, other miRNAs of the same family may show a compensatory effect. To test whether this is true in HT-29 cells, varying concentrations of a synthetic specific miRNA inhibitor (anti-miR-200c-3p) and negative control inhibitor were transfected into the cells (**Fig. 7C**). Expression of hsa-miR-200b-3p and hsa-miR-429 (both having the same seed sequence as hsa-miR-200c-3p) was assessed. Compared to transfection with the negative control, miRNA expression of hsa-miR-200b-3p and hsa-miR-429 significantly increased (FC = 1.9 and 2.3, respectively) in the cells transfected with 5 nM of anti-miR-200c-3p, while expression significantly decreased (FC = -2.2 and -1.9, respectively) after transfection with 20 nM. However, expression of both miR-200c family members was not significantly affected after transfection with 10 nM anti-miR-200c-3p (**Fig. 7C**). These results suggest that a concentration of 10 nM miRNA inhibitor does not alter the expression of miRNA family members.

To verify that hsa-miR-200c-3p directly targets the 3′UTR of *IL8* and *CDH11*, we co-transfected the luciferase vectors with a 10 nM of anti-miR-200c-3p or a negative control inhibitor in HT-29 cells. In cells transfected with the negative control, luciferase activity was decreased compared to the original luciferase vector, because of endogenous miRNAs. The miR-200c-3p inhibitor restored the relative luciferase activity of pGL3_IL8_WT and pGL3_CDH11_WT with 10% and 30% respectively (p<0.01), indicating the binding of hsa-miR-200c-3p to the target 3′UTRs. Luciferase activity of mutant vectors was not significantly different after transfection with anti-miR-200c-3p or negative control **(** **Fig. 7D**). These results indicate that *IL8* and *CDH11* are directly targeted by hsa-miR-200c-3p.

## Discussion

Recently, several papers have focused on investigating altered expression of miRNAs in IBD [36]–[38]. However, the majority of these studies only observed the expression of a limited amount of miRNAs. Moreover, there is a lack of studies integrating both miRNA and mRNA expression profiles in IBD. This study is the first whole-genome miRNA and mRNA expression microarray profiling in UC mucosa. Several studies have assumed that an inverse correlation between miRNA and mRNA expression levels may reflect miRNA – target mRNA relationship. Therefore, we performed a genome-wide analysis of both miRNA and mRNA expression in colonic mucosal UC biopsies. This correlation analysis reveals a useful approach to identify genes potentially targeted by dysregulated miRNAs. Furthermore, we found evidence that these miRNAs can directly target genes related to UC pathogenesis.

Hierarchical cluster analysis of both miRNA and mRNA expression profiles allowed us to clearly identify two different clusters related to the inflammatory load in the colonic mucosa. Comparative analysis between active UC patients and controls identified 51 differentially expressed miRNAs and 1288 differentially expressed annotated genes. More than half of these mRNAs are potentially targeted by one or more of the dysregulated miRNAs. Both the complete list of differentially expressed mRNAs as the subset of mRNAs which are potentially targeted by altered miRNAs, are mainly involved in immune-related functions. This result suggests a role of miRNAs in the regulation of gene expression in UC.

Our miRNA expression results identified several miRNAs that correspond with those found in previous studies comparing miRNA profiles in colonic mucosa of active UC patients *vs.* controls. The first study investigating this subject identified 11 miRNAs significantly differentially expressed in active UC mucosa *vs.* controls [13]. We could confirm the upregulation of hsa-miR-21-5p and the downregulation of hsa-miR-192-5p. Wu *et al.* [13] identified the chemokine macrophage inflammatory protein 2-α (*MIP-2α* or *CXCL2*) as target mRNA of hsa-miR-192-5p. Here, we confirm the strong increase of *MIP-2α* expression in active UC *vs.* controls (FC = 5.06), most likely due to the downregulation of hsa-miR-192-5p (FC = -2.98). Takagi *et al.* also observed an upregulation of hsa-miR-21-5p and hsa-miR-155-5p in active UC mucosa compared to controls [15]. We could confirm the upregulation of 2 miRNAs (hsa-miR-31-5p and hsa-miR-223-3p) out of 9 miRNAs that were identified as dysregulated in active UC mucosa by Fasseu *et al.* Lin *et al.* identified 4 miRNAs with an increased expression in UC, of which we could confirm the upregulation of hsa-miR-31-5p and hsa-miR-146a-5p. The authors propose hsa-miR-31-5p as a diagnostic biomarker of IBD to differentiate from its mimics, including IC [19]. Our study can't confirm this hypothesis because expression levels of hsa-miR-31-5p were elevated in both UC and IC patients compared to controls. Min *et al.* identified 68 miRNAs differentially expressed in active UC *vs.* controls [20]. Here, we could confirm the upregulation of hsa-let-7i-5p, hsa-miR-21-5p, hsa-miR-146a-5p and hsa-miR-155-5p, and the downregulation of hsa-miR-196b-5p, hsa-miR-200a-5p, hsa-miR-200a-3p, hsa-miR-200c and hsa-miR-378a-3p. In a cohort of pediatric UC patients, Zahm *et al.* identified 8 altered miRNAs in rectum of UC patients compared to controls [21]. Dysregulation of 7 of these miRNAs coincides with our results (upregulation of hsa-let-7i-5p, hsa-miR-21-5p and hsa-miR-146a-5p; downregulation of hsa-miR-192-5p, hsa-miR-194-5p and hsa-miR-200b-3p). Olaru *et al.* studied the miRNA expression in colonic biopsies of active IBD patients *vs.* IBD-associated dysplasia [39]. They identified 10 up- and 22 downregulated miRNAs of which 3 miRNAs were upregulated and 13 miRNAs were also downregulated in active UC *vs.* controls. The same authors also reported a linear increase in expression of hsa-miR-31-5p along the evolution of normal colon tissue, to IBD and to IBD-associated dysplasia [39]. Here, we confirm the strong increase in expression of hsa-miR-31-5p in active UC *vs.* controls. Iborra *et al.* reported hsa-miR-650 and hsa-miR-196b-5p as altered in active UC *vs.* inactive UC at tissue level [17]. In this study, we confirm the strong upregulation of hsa-miR-650 in active UC compared to both inactive UC (FC = 11.59) and controls (FC = 13.69) and a downregulation of hsa-miR-196b-5p in active UC *vs.* controls. Brest *et al.* demonstrated an upregulation of hsa-miR-196a/b-5p in the inflamed mucosa of CD patients [11]. Remarkably, both hsa-miR-196a-5p and hsa-196b-5p are downregulated in active UC *vs.* controls. Therefore, expression of miR-196a/b-5p might be useful to differentiate UC from CDc. According to published literature, few miRNAs are consistently dysregulated in active UC mucosa compared to controls. Although, miRNAs such as hsa-miR-21-5p, hsa-miR-31-5p or hsa-miR-155-5p are reported in several studies, and have potential as biomarker. However, in this study these miRNAs showed a similar increased expression in active UC as in active CDc and IC colonic tissue. Interestingly, we showed a downregulation of hsa-miR-200c-3p in active UC and of hsa-miR-196b-5p in both active and inactive UC mucosa compared to controls, while expression of both miRNAs is upregulated in inflammatory controls. Therefore, hsa-miR-200c-3p and/or hsa-miR-196b-5p show promise as a diagnostic biomarker of UC, although further verification is needed.

The functional role of multiple significantly different miRNAs has already been studied extensively. Three miRNAs (hsa-miR-155-5p, hsa-miR-21-5p, hsa-miR-146a-5p) together regulate inflammation through regulation of the Toll-like receptor (TLR) signaling pathway [40]. The inflammatory response is characterized by a continuous upregulation of hsa-miR-155-5p [8]. Furthermore, this miRNA directly targets mRNAs coding for several proteins involved in lipopolysaccharide (LPS) signaling while enhancing TNF-α translation [41]. Expression of hsa-miR-21-5p is important in the LPS-induced inflammatory response, because it has an essential role in the regulation of the pro- (NF-κB) as well as the anti-inflammatory (*IL-10*) response [42]. Also hsa-miR-146a-5p helps fine-tuning the immune response through a negative feedback regulation by direct targeting of *IRAK1* and *TRAF6*, two mediators of the TLR signaling pathway [9]. Involvement of this trio of miRNAs in active UC is confirmed as they are upregulated in the comparison *vs.* controls. Moreover, in our study was hsa-miR-155-5p the most significantly different miRNA (FDR = 2.22×10^−5^). Also other significantly different miRNAs have interesting properties related to the immune system. Epithelium-expressed hsa-miR-375 has an important role in epithelium immune system by regulating goblet cell differentiation [43]. Regulation of host gene expression by gut microbiota is mediated by hsa-miR-665 [44]. Hsa-miR-223-3p is an essential modulator of myeloid differentiation and has a role in IL-17-mediated inflammation [45], while hsa-miR-150-5p is upregulated during B- and T-cell maturation [46]. We confirm the dysregulation of these miRNAs in active UC and consequently their association with UC pathogenesis.

When comparing miRNA microarray expression levels of inactive UC patients and controls, we found no significant miRNA expression differences between inactive UC and controls. However, after measuring expression levels of hsa-miR-196b-5p using qRT-PCR, we found a significant difference between both groups. This could be explained by the fact that the statistical criteria used for defining significance by qRT-PCR (Mann-Whitney p-value <0.05) is less strict than the criteria used for microarray data significance (>2-fold change and FDR<0.05). Previous work has demonstrated significant differences in inactive UC patients *vs.* controls [16]. However, that study investigated the miRNA expression in non-inflamed tissue of UC patients with clinical and endoscopic active disease, whereas we examined UC patients in clinical, endoscopic and histologic remission. Also other publications report significant differences in miRNA expression in inactive UC mucosa *vs.* controls [13], [18]. Though, no validated score for histological inflammation was applied to the biopsies in these studies. Therefore, we hypothesize the reason for these dissimilarities could be the more strict criteria for patient selection or a difference in histopathological grades of UC activity.

Correlation analysis between miRNA and the predicted target mRNA expression levels showed that 4 of the top 10 most significantly inversely correlated miRNA – target mRNA pairs belong to hsa-miR-200c-3p. Of interest, out of the four predicted target mRNAs are 3 UC susceptibility genes (*IL8*, *LRRK2*, *CALU*) and the fourth gene is related to the intestinal barrier function (*CDH11*). We confirmed the significant downregulation of hsa-miR-200c-3p and significant upregulation of *IL8* and *CDH11* in active UC *vs.* controls using qRT-PCR. Hsa-miR-200c-3p is known to be involved in innate immunity by regulating the efficiency of TLR4 signaling through the MyD88-dependent pathway [47]. Additionally, members of the miR-200 family are involved in the epithelial to mesenchymal transition (EMT) by regulating the E-cadherin transcriptional repressors *ZEB1* and *SIP1* both in cancer [48], [49]and in IBD [50]. One miRNA of this family, hsa-miR-200b-3p, is proposed as a diagnostic serum marker for fibrosis in Crohn's disease. Chen *et al.* suggest the potential role of hsa-miR-200b-3p in inhibiting EMT and promoting the proliferation of intestinal epithelial cells. Moreover, it could protect intestinal epithelial cells from fibrogenesis *in vitro* [50], [51].

UC is characterized by a strong increase of *IL8*, a powerful neutrophil chemoattractant and activator produced by endothelial cells, lamina propria mononuclear cells and epithelial cells [52]. *IL8* is a UC susceptibility gene, recently discovered in the latest meta-analysis of IBD GWAS studies [35]. It is a key marker in colonic inflammation, and therefore seems to be a reliable biomarker, closely related to disease activity [53]. Interestingly, it has been shown that *IL8* expression is indirectly regulated by hsa-miR-200c-3p by targeting IKBKB [54]. Also other miRNAs are involved in *IL8* regulation like hsa-miR-155-5p by lowering SHIP1 expression [55], and hsa-miR-146a/b-5p by negatively regulating NF-κB activity [56]. In this study, we demonstrated that in UC hsa-miR-200c-3p also directly regulates *IL8* expression. This result is consistent with previous localization studies in normal and UC mucosa, where it has been shown that hsa-miR-200c-3p and IL8 are expressed in colonic epithelial cells [49], [57].

Previous microarray profiling studies in UC have already implicated a role for *CDH11*, a type II classical cadherin that mediates fibroblast cell-cell adhesion, in UC pathogenesis. Costello *et al.* have shown a significant upregulation of *CDH11* in IBD, but not in inflamed non-IBD tissue. It is presumed this member of the cadherin family could be involved in restructuring processes in the intestinal mucosa [58]. Here, we confirm the specific role this gene might have in active UC by the fact that *CDH11* expression is not dysregulated in inflammatory controls compared to controls. Using an integrated analysis of miRNA and mRNA expression in breast cancer, Luo *et al.* recently identified *CDH11* as a target gene of hsa-miR-200c-3p [59]. In the present study, we demonstrated that *CDH11* is also targeted by hsa-miR-200c-3p in colonic epithelial cells. Downregulation of *CDH11* could serve as an interesting therapeutic goal in UC, potentially by the use of a mimic of hsa-miR-200c-3p. In a dextran sodium sulfate-colitis mouse model, *CDH11* deficient mice are significantly protected from severe colitis compared to wild-type mice [60]. This suggests that targeting CDH11 may be of interest in IBD. In pulmonary fibrosis, expression of *CDH11* is also increased in wound healing and fibrotic skin. Anti-CDH11-neutralizing monoclonal antibodies (mAb) successfully treated established pulmonary fibrosis in mice. Thus, *CDH11* is a mediator in EMT and the development of pulmonary fibrosis [61]. Furthermore, in rheumatoid arthritis *CDH11* modulates synovial fibroblasts to evoke inflammatory factors and targeting *CDH11* by mAb directed against *CDH11* significantly reduced inflammation [62]. Intestinal fibrosis can occur in both CD as UC. Inflammation of the (sub)mucosal layers in UC can induce fibrosis through EMT [63]. Being a mediator of EMT in the development of organ fibrosis and a contributor to the inflammatory process in rheumatoid arthritis, it could be hypothesized that *CDH11* is a newly identified candidate therapeutic target for fibrosis in IBD.

As a potential therapeutic, miRNAs have inherent advantages: they are evolutionary conserved and due to their small size miRNAs are more resistant to degradation. In a pathological condition, upregulated miRNAs can be decreased by potent oligonucleotides, ‘antagomirs’, that inhibit their miRNA targets by binding with high affinity and specificity. Downregulated miRNAs can be increased by using synthetic oligonucleotide miRNA mimics or miRNA expression gene vectors [64]. Currently, several clinical trials are testing the efficacy of these therapies. Recently, a phase 2a study for the use of miravirsen, an antagomir that sequesters miR-122-5p, for treatment of hepatitis C infection was successfully completed [65]. In chronic inflammatory diseases, miR-155-5p antagonists show possible therapeutic value by modulating activation of macrophages and the number of circulating granulocytic cells [66]. Potentially, treatment with a synthetic mimic of miR-200c-3p in IBD could decrease inflammation by counteracting the inflammatory action of *IL8*, or by down-modulating the NF-κB response after TLR4 ligation. Moreover, this treatment could inhibit EMT and prevent fibrosis in the pathogenesis of IBD.

In conclusion, this study represents an integrated analysis of miRNA and mRNA expression in colonic mucosal UC biopsies. We identified a number of miRNAs that are differentially expressed between active UC and controls, supporting the hypothesis that altered expression of miRNAs plays a role in the expression of immune-related (e.g. *IL8*) and barrier-related (e.g. *CDH11*) genes in inflamed UC mucosa. Integrated analysis of miRNA and gene expression profiles revealed potential targets, such as hsa-miR-200c-3p, for use of miRNA mimics as therapeutics.

## Supporting Information

S1 Fig Venn diagram of the overlap of mRNA profiles in comparative analyses between (in)active UC and controls. The differentially expressed mRNAs in the comparative analyses between active UC, inactive UC and controls are depicted in three overlapping circles.
(TIF)

S2 Fig Biological functions associated to active UC. Bar chart representing the top 10 most significant biological functions that were associated with the 1543 significantly differentially expressed probe sets in active UC *vs.* controls. The functional categories are displayed along the x-axis and the y-axis indicates the significance score.
(TIF)

S3 Fig Biological functions associated to inactive UC. Bar chart representing the top 10 most significant biological functions that were associated with the significantly differentially expressed probe sets in inactive UC *vs.* controls. The functional categories are displayed along the x-axis and the y-axis indicates the significance score.
(TIF)

S4 Fig Biological functions associated to predicted target mRNAs of dysregulated miRNAs in active UC. Bar chart representing the top 10 most significant biological functions that were associated with the 3328 pairs of miRNAs and their predicted target mRNAs that are altered in active UC *vs.* controls. The functional categories are displayed along the x-axis and the y-axis indicates the significance score.
(TIF)

S1 Table A list of all genes focused on in the present study because of clinical interest in UC. The list contains 1227 UC susceptibility genes, 91 AMP proteins, 70 CAMs and 223 genes related to intestinal barrier.
(XLSX)

S2 Table miRNAs differentially expressed in active UC as compared with normal controls. A list of the 51 significantly differentially expressed miRNAs in active UC *vs.* controls with their corresponding fold change (FC) values. A significant miRNA has a false discovery rate (FDR)<5% and FC>2. miRNAs that were also significantly differentially expressed in active UC *vs.* inactive UC are indicated in bold.
(XLSX)

S3 Table mRNAs differentially expressed in active UC as compared with normal controls.
(XLSX)

S4 Table Selected inversely correlated significantly differentially expressed miRNAs and their predicted target mRNAs. miRNAs belonging to the same family are grouped together.
(XLSX)

S5 Table List of all 318 significantly inversely correlated miRNA-mRNA pairs.
(XLSX)
