# Supplementary figures and images for: Integrated miRNA and mRNA Expression Profiling in Inflamed Colon of Patients with Ulcerative Colitis
Source: PLoS One. 2014 Dec 29;9(12):e116117. doi: 10.1371/journal.pone.0116117 (PMC4278881; doi:10.1371/journal.pone.0116117)

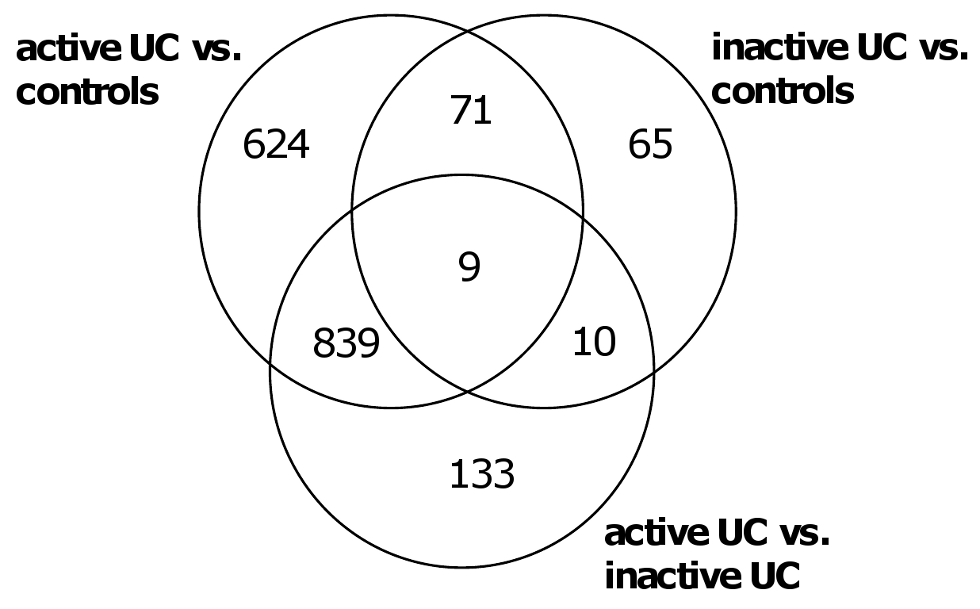

Supplement: S1 Fig — Venn diagram of the overlap of mRNA profiles in comparative analyses between (in)active UC and controls. The differentially expressed mRNAs in the comparative analyses between active UC, inactive UC and controls are depicted in three overlapping circles. (TIF) [file pone.0116117.s001.tif]

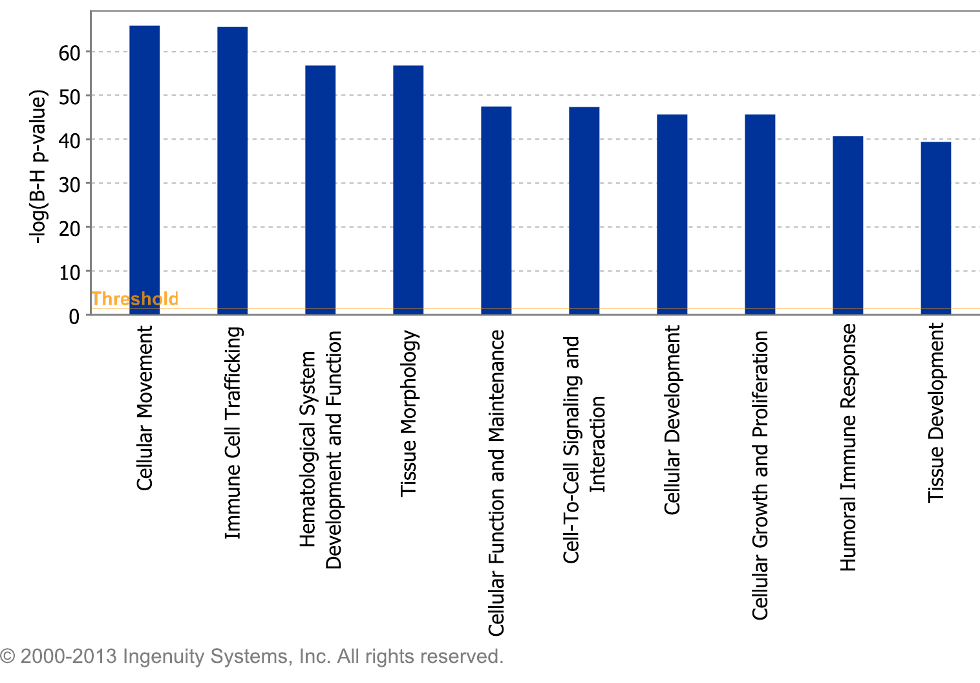

Supplement: S2 Fig — Biological functions associated to active UC. Bar chart representing the top 10 most significant biological functions that were associated with the 1543 significantly differentially expressed probe sets in active UC vs. controls. The functional categories are displayed along the x-axis and the y-axis indicates the significance score. (TIF) [file pone.0116117.s002.tif]

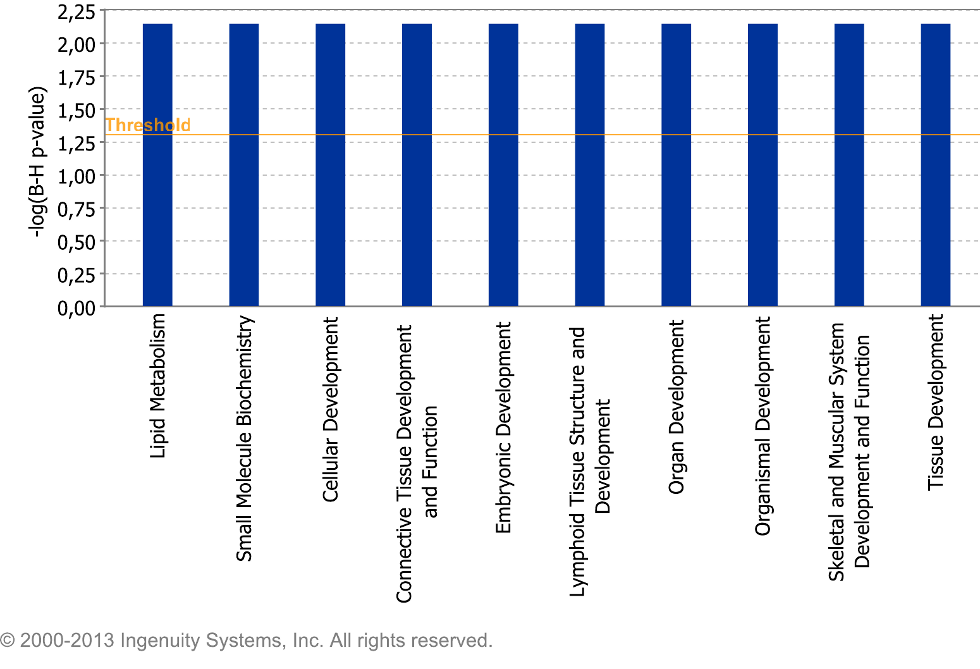

Supplement: S3 Fig — Biological functions associated to inactive UC. Bar chart representing the top 10 most significant biological functions that were associated with the significantly differentially expressed probe sets in inactive UC vs. controls. The functional categories are displayed along the x-axis and the y-axis indicates the significance score. (TIF) [file pone.0116117.s003.tif]

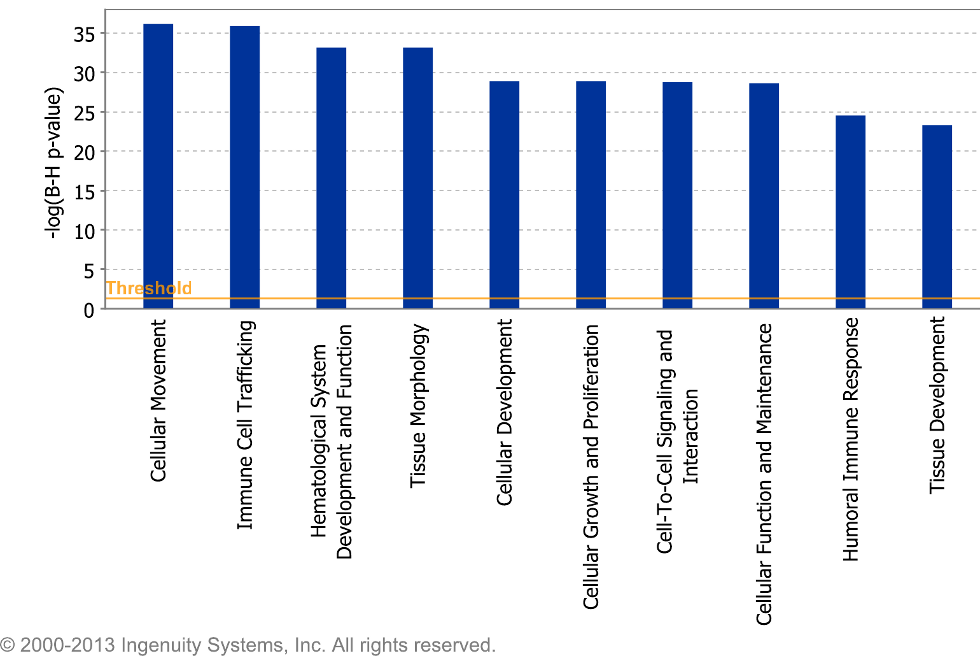

Supplement: S4 Fig — Biological functions associated to predicted target mRNAs of dysregulated miRNAs in active UC. Bar chart representing the top 10 most significant biological functions that were associated with the 3328 pairs of miRNAs and their predicted target mRNAs that are altered in active UC vs. controls. The functional categories are displayed along the x-axis and the y-axis indicates the significance score. (TIF) [file pone.0116117.s004.tif]
